# Unraveling the Role of Proteinopathies in Parasitic Infections

**DOI:** 10.3390/biomedicines13030610

**Published:** 2025-03-03

**Authors:** Mikołaj Hurła, Damian Pikor, Natalia Banaszek-Hurła, Alicja Drelichowska, Jolanta Dorszewska, Wojciech Kozubski, Elżbieta Kacprzak, Małgorzata Paul

**Affiliations:** 1 Department of Tropical and Parasitic Diseases, Central University Hospital, Przybyszewskiego 49, 61-701 Poznan, Poland; 2 Department of Internal Medicine, University of Medical Sciences, Przybyszewskiego 49, 60-355 Poznan, Poland; 3 Laboratory of Neurobiology, Department of Neurology, Poznan University of Medical Sciences, 60-355 Poznan, Poland; 4 Student Scientific Society of Poznan, University of Medical Sciences, 60-806 Poznan, Poland; 5 Chair and Department of Neurology, Poznan University of Medical Sciences, 60-355 Poznan, Poland

**Keywords:** proteinopathies, protein misfolding, parasitic infections, host–parasite interactions, unfolded protein response (UPR), proteostasis

## Abstract

Proteinopathies, characterized by the misfolding, aggregation, and deposition of proteins, are hallmarks of various neurodegenerative and systemic diseases. Increasingly, research has highlighted the role of protein misfolding in parasitic infections, unveiling intricate interactions between host and parasite that exacerbate disease pathology and contribute to chronic outcomes. The life cycles of parasitic protozoa, including Plasmodium, Toxoplasmosis, and Leishmania species, are complicated and involve frequent changes between host and vector environments. Their proteomes are severely stressed during these transitions, which calls for highly specialized protein quality control systems. In order to survive harsh intracellular conditions during infection, these parasites have been demonstrated to display unique adaptations in the unfolded protein response, a crucial pathway controlling endoplasmic reticulum stress. In addition to improving parasite survival, these adaptations affect host cell signaling and metabolism, which may jeopardize cellular homeostasis. By causing oxidative stress, persistent inflammation, and disturbance of cellular proteostasis, host–parasite interactions also contribute to proteinopathy. For instance, Plasmodium falciparum disrupts normal protein homeostasis and encourages the accumulation of misfolded proteins by influencing host redox systems involved in protein folding. In addition to interfering with host chaperone systems, the parasitic secretion of effector proteins exacerbates protein misfolding and aggregate formation. Autophagy, apoptosis regulation, organelle integrity, and other vital cellular processes are all disrupted by these pathological protein aggregates. Long-term misfolding and aggregation can cause irreversible tissue damage, which can worsen the clinical course of illnesses like visceral leishmaniasis, cerebral malaria, and toxoplasmosis. Treating parasite-induced proteinopathies is a potentially fruitful area of therapy. According to recent research, autophagy modulators, proteasome enhancers, and small-molecule chaperones may be repurposed to lessen these effects. Pharmacological agents that target the UPR, for example, have demonstrated the ability to decrease parasite survival while also reestablishing host protein homeostasis. Targeting the proteins secreted by parasites that disrupt host proteostasis may also offer a novel way to stop tissue damage caused by proteinopathies. In conclusion, the intersection of protein misfolding and parasitic infections represents a rapidly advancing field of research. Dissecting the molecular pathways underpinning these processes offers unprecedented opportunities for developing innovative therapies. These insights could not only transform the management of parasitic diseases but also contribute to a broader understanding of proteinopathies in infectious and non-infectious diseases alike.

## 1. Introduction

Parasitic infections remain a significant global health challenge, contributing to millions of deaths and disabilities annually [1]. While the direct pathological effects of these infections are well-documented, emerging research has revealed a more insidious mechanism by which parasites disrupt host cellular function: the induction of protein misfolding and aggregation [2,3]. These processes, collectively referred to as proteinopathies, are typically associated with neurodegenerative diseases such as Alzheimer’s and Parkinson’s [4]. However, recent studies have demonstrated that not only infectious agents but also parasitic protozoa, including Plasmodium [5,6], Toxoplasma [7,8], and Leishmania species [9,10], can also trigger protein misfolding in host cells, leading to severe clinical outcomes such as cerebral malaria, systemic or neuro toxoplasmosis, and visceral leishmaniasis [11,12,13]. Proteinopathies are characterized by the accumulation of misfolded proteins, which disrupt cellular homeostasis and contribute to tissue damage [14]. In the context of parasitic infections, these processes are not merely bystander effects but are actively exploited by parasites to create a favorable environment for their survival and replication [15]. By manipulating host protein quality control systems [16], inducing oxidative stress [17], and disrupting cellular signaling pathways [18], parasites exacerbate disease pathology and contribute to chronic conditions [19]. This intricate interplay between host and parasite highlights the complexity of parasitic infections and underscores the need for innovative therapeutic strategies that target protein misfolding and aggregation [20]. The study of parasite-induced proteinopathies not only enhances our understanding of infectious diseases but also provides valuable insights into the mechanisms underlying non-infectious proteinopathies. For instance, the molecular pathways disrupted by Plasmodium falciparum in cerebral malaria share striking similarities with those observed in neurodegenerative disorders, offering a unique lens through which to explore the pathogenesis of conditions like Alzheimer’s and Parkinson’s diseases [4,14]. Similarly, Toxoplasma gondii-induced neurodegeneration and the Leishmania-mediated disruption of autophagy provide models for studying the role of inflammation, mitochondrial dysfunction, and immune responses in protein misfolding and aggregation [9,12]. Immunosuppression, such as that induced by chemotherapy or underlying conditions like HIV/AIDS, plays a significant role in worsening the prognosis of parasitic infections like toxoplasmosis and leishmaniasis [7,10]. In immunocompromised individuals, the host’s ability to control parasitic replication and mitigate proteinopathic damage is severely impaired, leading to more aggressive disease progression and higher mortality rates [19]. For example, in *Toxoplasma gondii* infections, immunosuppression can result in the reactivation of latent infections, leading to severe toxoplasmic encephalitis characterized by widespread protein misfolding and neuronal damage [8]. Similarly, in visceral leishmaniasis, immunosuppressed patients often experience exacerbated protein aggregation and organ dysfunction due to the inability of the immune system to regulate parasite burden and associated cellular stress responses [10]. This population-level vulnerability underscores the importance of considering host immune status in the management and treatment of parasitic infections. This manuscript aims to explore the mechanisms by which parasitic infections induce proteinopathies, the clinical implications of these processes, and their potential to inform therapeutic strategies for both infectious and non-infectious diseases. By examining the intersection of parasitology and protein homeostasis, we hope to shed light on novel pathways for intervention and contribute to the broader understanding of proteinopathies in human health and disease. The inclusion of immunosuppression as a critical factor in disease severity further emphasizes the need for tailored therapeutic approaches that address both the parasitic infection and the host’s immune status, particularly in vulnerable populations.

## 2. Protein Misfolding Induced by Parasites

Protein misfolding is a critical cellular event that can lead to the accumulation of toxic protein aggregates, the disruption of cellular homeostasis, and ultimately cell death. Intracellular protozoan parasites, such as Plasmodium, Toxoplasma, and Leishmania, have evolved complex mechanisms to manipulate host cell processes, including the induction of protein misfolding. These parasites exploit the host’s protein quality control (PQC) machinery to create a favorable environment for their survival, replication, and immune evasion. This section explores how Plasmodium, Toxoplasma, and Leishmania induce protein misfolding in host cells, the consequences of this misfolding, and the potential therapeutic implications (Table 1).

### 2.1. Plasmodium Species

Plasmodium species, particularly Plasmodium falciparum, are responsible for malaria, a life-threatening disease that affects millions worldwide. During its life cycle, Plasmodium invades and replicates within host red blood cells (RBCs), causing significant alterations in the host cell’s proteome. The parasite exports numerous proteins into the host cytoplasm, many of which are intrinsically disordered or contain low-complexity regions, making them prone to misfolding and aggregation [1,2]. One of the key mechanisms by which Plasmodium induces protein misfolding in host cells is through the export of virulence factors such as Plasmodium falciparum erythrocyte membrane protein 1 (PfEMP1). PfEMP1 is a major virulence factor that mediates the cytoadherence of infected RBCs to endothelial cells, allowing the parasite to evade splenic clearance [3]. However, the export and proper folding of PfEMP1 require the assistance of parasite-encoded heat shock proteins (HSPs), such as HSP70 and HSP40, which are also exported into the host cytoplasm [4,5]. The interaction between PfEMP1 and host cytoskeletal proteins, such as spectrin, can lead to the destabilization of the RBC cytoskeleton, resulting in protein misfolding and aggregation [6]. This destabilization not only facilitates parasite survival but also contributes to the pathogenesis of severe malaria, including cerebral malaria and placental malaria [7]. Moreover, Plasmodium induces oxidative stress in infected RBCs, which further exacerbates protein misfolding. The parasite digests hemoglobin within the parasitophorous vacuole, releasing heme as a byproduct. Heme is highly reactive and can generate reactive oxygen species (ROS), leading to oxidative damage in host proteins and lipids [8]. The accumulation of misfolded proteins in infected RBCs triggers the host’s unfolded protein response (UPR), a cellular stress response related to the endoplasmic reticulum (ER) [9]. However, Plasmodium has evolved mechanisms to subvert the host UPR, ensuring its survival despite the accumulation of misfolded proteins [10]. The parasite also exploits the host’s UPS to degrade misfolded proteins. Plasmodium exports proteins that interact with host ubiquitin ligases, leading to the ubiquitination and subsequent degradation of host proteins involved in immune responses [11]. This manipulation of the host UPS not only aids in immune evasion but also disrupts host cell homeostasis, further promoting protein misfolding and aggregation [12]. In addition to these mechanisms, Plasmodium also induces protein misfolding through the disruption of host cell calcium homeostasis. The parasite exports proteins that interact with host calcium channels, leading to an increase in intracellular calcium levels [13]. Elevated calcium levels can disrupt the folding of host proteins, particularly those involved in signal transduction and cytoskeletal organization [14]. This disruption of calcium homeostasis not only contributes to protein misfolding but also facilitates parasite egress from infected RBCs [15]. In conclusion, Plasmodium induces protein misfolding in host cells through multiple mechanisms, including the export of virulence factors, induction of oxidative stress, manipulation of the host UPR and UPS, and disruption of calcium homeostasis, all of which contribute to its survival and pathogenesis.

### 2.2. Toxoplasma gondii

*Toxoplasma gondii* is an obligate intracellular parasite that causes toxoplasmosis, a disease with severe consequences for immunocompromised individuals and children with congenital infection. As with other intracellular parasites, Toxoplasma employs strategies that disrupt host cellular processes, yet its approach has distinct nuances. Like Plasmodium, it induces protein misfolding in host cells as part of its survival strategy [16]. After invading host cells, the parasite resides within a parasitophorous vacuole (PV) and secretes a diverse array of effector proteins that interfere with host cell signaling and homeostasis. In a unique twist, Toxoplasma relies on the secretion of dense granule proteins (GRAs) to modify the PV membrane and attract host organelles such as mitochondria and the ER [17]. This close organelle association can mislocalize host proteins, causing them to misfold and aggregate [18]. For example, a disruption of the host ER by Toxoplasma infection results in the accumulation of misfolded proteins and the activation of the unfolded protein response (UPR), although the parasite simultaneously modulates the UPR to favor its persistence [19,20]. Furthermore, Toxoplasma generates reactive oxygen species (ROS) during its metabolism, thereby inducing oxidative stress that damages host proteins and lipids [25]. The resulting buildup of oxidized proteins activates host antioxidant defenses, upregulating heat shock proteins (HSPs) and the ubiquitinproteasome system (UPS) [26] while the parasite further influences these processes by secreting HSP70 into the host cytoplasm, which interacts with host proteins and contributes to misfolding [27,28]. A particularly notable aspect of its strategy is the manipulation of autophagy. Although autophagy normally degrades damaged organelles and misfolded proteins through autophagosome formation [29], Toxoplasma not only triggers autophagy to secure nutrients [30] but also secretes proteins that block autophagosome–lysosome fusion, ensuring that misfolded proteins are only partially degraded, thereby sustaining a nutrient-rich environment for the parasite [31]. In addition, Toxoplasma disturbs host cell calcium homeostasis by exporting proteins that interfere with calcium channels, leading to increased intracellular calcium levels that further destabilize protein folding and facilitate parasite egress [1,3,6].

### 2.3. Leishmania Species

Leishmania species are intracellular parasites that cause leishmaniasisa disease with a wide range of clinical manifestations, including cutaneous, mucocutaneous, and visceral forms [15]. While Leishmania, like Toxoplasma gondii, invade host cells (in this case, primarily macrophages) and reside within parasitophorous vacuoles, its strategies to manipulate host protein folding share similarities with, yet also diverge from, those of Toxoplasma. Instead of using dense granule proteins, Leishmania predominantly secretes exosomes small extracellular vesicles that carry a mix of proteins, lipids, and nucleic acidsto deliver its effector molecules [10]. These exosomes are enriched with virulence factors such as HSP70 and HSP90, which modulate the host immune response and disturb protein folding in a manner comparable to the actions of Toxoplasma’s secreted factors [4,9]. Similar to Toxoplasma, Leishmania also induces oxidative stress by generating ROS during its metabolic processes, which leads to oxidative damage in host proteins and lipids [16]. This stress in turn activates the host’s antioxidant defenses, including the upregulation of HSPs and activation of the UPS [8]. However, while both parasites utilize HSP70 to manipulate host responses, Leishmania incorporates its HSP70 within exosomal cargo, emphasizing a distinct mode of delivery [11,12]. In terms of autophagy, Leishmania mirrors Toxoplasma by inducing autophagy to degrade misfolded proteins and liberate nutrients that support its growth [2,5]. However, it too produces proteins that inhibit the fusion of autophagosomes with lysosomes, a strategy that, despite its similarity to Toxoplasma’s approach, appears to be finetuned differently in timing and regulatory control [14]. Additionally, Leishmania disrupts host calcium homeostasis by exporting proteins that interact with host calcium channels, resulting in elevated intracellular calcium levels that not only disturb protein folding but also facilitate parasite egress from infected cells [7,13,20].

## 3. Therapeutic Approaches and Opportunities

Parasite-induced proteinopathies present unique challenges and opportunities for therapeutic intervention. Addressing these conditions requires a multifaceted approach targeting various aspects of the host’s and parasite’s protein quality control systems (Table 2).

### 3.1. Autophagy Modulators and Proteasome Enhancers for Managing Proteostasis

Cellular health is largely dependent on the preservation of proteostasis, which is the delicate balance between protein synthesis, folding, and degradation. When this balance is upset, misfolded proteins can build up and become a defining feature of many diseases, including neurodegenerative conditions. In order to restore proteostasis, recent developments have shed light on therapeutic approaches that increase proteasome activity and modify autophagy. One essential mechanism for the breakdown of damaged or misfolded proteins is the UPS. It has been shown that 26S proteasome activation promotes the degradation of proteins linked to neurodegenerative diseases. For instance, it has been demonstrated that cGMP-mediated PKG activation increases protein clearance by stimulating the 26S proteasome [37,38]. Furthermore, small molecules that can activate the 20S proteasome have shown promise as therapeutic agents for addressing proteins that are intrinsically disordered and linked to proteotoxic diseases [39,40]. The maintenance of proteostasis also depends on autophagy, a lysosome-dependent degradation pathway. HSPB8 is a key component of the Chaperone-Assisted Selective Autophagy (CASA) complex, which specifically targets misfolded proteins for autophagic degradation. One tactic to lessen diseases associated with protein misfolding is the therapeutic modification of CASA complex components [41]. Additionally, new research has revealed arginine to be a novel autophagy regulator, indicating that autophagic processes may be influenced by amino acid signaling pathways [42]. Because autophagy and the UPS interact so intricately, a comprehensive therapeutic strategy that addresses both systems is required. In experimental models of neurodegenerative diseases, for example, it has been demonstrated that improving autophagic pathways reduces proteotoxic stress [43]. Furthermore, the importance of chaperone-mediated autophagy in maintaining proteostasis is highlighted by the protective function of small heat shock proteins in reducing neurodegeneration [44].

### 3.2. Chaperones as Misfolded Protein Stabilizers

To maintain correct protein folding, prevent aggregation, and preserve cellular homeostasis under both physiological and stress conditions, chaperones—key mediators of cellular proteostasis—have emerged as essential stabilizers of misfolded proteins. Chaperones, such as the HSP families, fulfil a diverse array of functions in cellular processes and stress responses, which are underscored by their dynamic and sophisticated mechanisms [45]. A critical aspect of their operation lies in their ability to recognize and bind exposed hydrophobic residues on misfolded proteins, thereby preventing these unstable regions from aggregating and enabling proper refolding or degradation via collaborative systems such as the UPS [36]. This role is exemplified by molecular chaperones such as HSP70 and HSP90, whose ATP-dependent cycles of substrate binding and release are meticulously regulated by co-chaperones [46,47]. HSP70, in particular, has been extensively studied for its ability to stabilize unfolded proteins and facilitate their delivery to the proteasome via co-chaperones such as BAG-1 and BAG-6, which also serve as ubiquitin-like domain proteins connecting misfolded proteins to degradation pathways [48,49]. Nucleotide exchange factors (NEFs) and J-domain proteins (JDPs) further enhance the specificity and efficacy of these processes by modulating the conformational states of HSP70 to optimize substrate interaction [50,51]. The recently identified mitochondria-mediated proteostasis mechanism, termed MAGIC (mitochondria as guardians in the cytosol), introduces an additional dimension to the role of chaperones in managing misfolded proteins. This pathway facilitates the import of misfolded cytosolic proteins into mitochondria for degradation, particularly under heat shock conditions. This mechanism highlights the adaptive versatility of the cellular proteostasis network, complementing the proteasome in alleviating proteotoxic stress through mitochondrial import and subsequent proteolysis [52]. Studies further underscore the critical reliance of this process on ATP hydrolysis and the central role of the mitochondrial import machinery, including the TOM/TIM complexes, thus emphasising the intricate interplay between cellular energy status and protein quality control [53,54]. Parasitic diseases such as leishmaniasis and malaria provide compelling examples of how chaperones are indispensable to pathogen survival and virulence. In Leishmania, heat shock proteins like HSP70 and HSP83 are critical for adaptation to the hostile environments of mammalian macrophages. These chaperones assist in protein folding, mitigate damage from reactive oxygen species, and stabilize key virulence factors necessary for survival within host cells. The reliance of Leishmania on its chaperone network highlights their potential as targets for therapeutic intervention [32]. Similarly, in Plasmodium falciparum, the causative agent of malaria, chaperones play pivotal roles in ensuring proteostasis during its complex lifecycle stages. HSP70 and HSP90 are instrumental in maintaining the parasite’s proteome under the febrile conditions of the human host, as well as during transmission between the mosquito vector and mammalian host [33,55]. A disruption of these systems by compounds such as violacein has been shown to collapse protein homeostasis, resulting in parasite death across multiple lifecycle stages [21,56]. It is increasingly recognised that chaperone dysfunction contributes to the pathogenesis of numerous illnesses, including neurodegenerative disorders such as Parkinson’s and Alzheimer’s diseases [57]. These conditions are typified by the accumulation of protein aggregates due to impaired proteostasis. The therapeutic potential of chaperones is underscored by their capacity to disaggregate or target these misfolded proteins for degradation. Additionally, the pharmacological modulation of chaperone activity, including the use of small-molecule activators or inhibitors, constitutes a rapidly advancing field. For instance, inhibitors targeting HSP90 have shown promise in destabilizing oncogenic client proteins, thereby impeding cancer cell proliferation [58,59]. Innovative therapeutic strategies for parasitic diseases have also capitalized on the pivotal role of chaperones in proteostasis. The natural compound violacein exemplifies this approach by disrupting the chaperone systems of Plasmodium falciparum, resulting in the collapse of protein homeostasis and exhibiting multistage antiplasmodial activity. This illustrates the potential of targeting parasite-specific chaperone networks to combat drug resistance and improve treatment outcomes [34]. Beyond stabilizing proteins, molecular chaperones are integral to broader cellular functions, including signalling, stress response, and inter-organelle communication. For instance, the mitochondrial and UPS pathways collaborate to resolve proteotoxic stress, with chaperones acting as essential intermediaries [22,60,61]. The integrative nature of these networks is further exemplified by co-chaperones such as CHIP, which combines chaperone and E3 ubiquitin ligase activities, thereby orchestrating the fate of misfolded proteins [62,63,64]. In conclusion, chaperones are indispensable to cellular health, serving as stabilizers of misfolded proteins and orchestrators of intricate proteostasis networks. Their roles in health and disease underscore their potential as therapeutic targets. Advancing our understanding of the mechanistic complexities of chaperone systems promises to unlock novel strategies for addressing proteostasis-related diseases, from neurodegeneration to infectious conditions, marking a significant frontier in biomedical research.

### 3.3. Targeting Parasite-Specific UPR Pathways to Impair Parasite Survival

A vital adaptation for Apicomplexan parasites and related kinetoplastids such as Leishmania spp., the UPR is a basic mechanism for preserving endoplasmic reticulum (ER) homeostasis during proteotoxic stress. To survive the various stresses imposed by host immune responses, such as oxidative bursts, febrile episodes, and nutrient deprivation, these pathogens depend on UPR pathways [65,66] Utilizing UPR components unique to parasites and different from those of their hosts highlights the therapeutic potential of this pathway in the fight against diseases like leishmaniasis, toxoplasmosis, and malaria.

### 3.4. Parasite-Specific UPR Pathways in Apicomplexa

The UPR in Apicomplexa is a highly specialized and essential mechanism that enables these intracellular parasites to withstand the hostile and dynamic conditions of the host cell environment. This intricate system integrates molecular chaperones, proteasomal degradation pathways, and stress signalling mechanisms to maintain protein homeostasis during endoplasmic reticulum (ER) stress, while also promoting parasite growth, replication, and pathogenesis. Among these components, the ER-resident chaperone BiP (PfHSP70-2) in Plasmodium falciparum is a key player, facilitating protein folding, preventing the aggregation of misfolded proteins, and participating in the ER-associated degradation (ERAD) pathway to remove damaged proteins [67]. PfHSP70-2 also plays a crucial role in the export of virulence factors by interacting with the exported protein-interacting complex (EPIC), ensuring the trafficking of PfEMP1 to the host erythrocyte membrane. PfEMP1 is instrumental in immune evasion, as it enables cytoadherence to endothelial cells and protects infected erythrocytes from splenic filtration [34]. Structural differences between PfHSP70-2 and its human homolog, such as a GGMP repeat region in the substrate-binding domain, enhance its ATPase activity and substrate affinity, making it a viable target for selective inhibition. Small molecules that disrupt PfHSP70-2 function can selectively impair parasite proteostasis, inducing lethal proteotoxic stress while sparing host cells [23,24,68]. Another pivotal component of the UPR in P. falciparum is the phosphorylation of eukaryotic initiation factor 2α (eIF2α), a key regulatory step mediated by the parasite-specific kinase PK4. This process selectively enhances the translation of stress-response proteins while suppressing global protein synthesis to alleviate ER stress [69,70]. This dual functionality enables the parasite to conserve resources and adapt to febrile episodes induced by host immune responses. Since PK4 is unique to P. falciparum, it represents a particularly promising therapeutic target. Preclinical studies demonstrate that inhibitors of PK4 or its downstream pathways exhibit potent antiparasitic effects, reducing parasite survival under heat stress without compromising host cell viability [71]. In Toxoplasma gondii, the ER-resident chaperone TgHSP70, homologous to PfHSP70-2, supports protein folding, ERAD, and the transition between tachyzoite and bradyzoite stages, which is critical for chronic infection persistence. Like PfHSP70-2, TgHSP70 contains a GGMP repeat region that boosts ATPase activity and facilitates selective inhibitor binding. The chemical inhibition of TgHSP70 significantly disrupts replication and persistence, underscoring its therapeutic potential [10,72]. Furthermore, T. gondii activates its UPR via a PERK-like kinase (TgIF2K-A), which phosphorylates Tg-eIF2α to suppress global protein synthesis while promoting stress-specific protein translation. This adaptation supports latency and enhances survival, highlighting TgIF2K-A as another promising target for drug development [73]. Unlike most eukaryotes, Apicomplexa lack heat shock transcription factors (HSFs) and instead rely on Apetala2 (AP2)-domain transcription factors to regulate UPR-related genes. In T. gondii, these AP2 factors orchestrate the expression of key molecular chaperones such as HSP70 and HSP90 during both acute and latent infections, with their evolutionary divergence making them attractive candidates for highly specific therapeutic targeting. UPS plays a complementary role in maintaining proteostasis by degrading damaged or misfolded proteins. Both T. gondii and P. falciparum possess unique E3 ligases and deubiquitinating enzymes (DUBs) that differ structurally and functionally from their human counterparts. Proteasome inhibitors have shown strong efficacy in disrupting protein homeostasis in these parasites, leading to toxic protein aggregation and impaired replication. These inhibitors provide a promising approach to overcoming resistance to existing antiparasitic drugs [74,75]. In addition to intrinsic UPR pathways, T. gondii manipulate host ER stress pathways, enhancing cytoskeletal remodeling, motility, and the dissemination of infected cells. Such manipulations also modulate host immune responses through the IRE1 and PERK pathways, facilitating parasite survival and propagation. Targeting these host–parasite interactions offers an innovative therapeutic strategy for toxoplasmosis [76,77]. In conclusion, the UPR in Apicomplexa is indispensable for parasite survival and pathogenesis, presenting a prime target for intervention. By targeting parasite-specific UPR components, including TgHSP70, eIF2α kinases, AP2 transcription factors, and the UPS, next-generation antiparasitic therapies could precisely and effectively disrupt critical survival mechanisms.

### 3.5. Parasite-Specific UPR Pathways in Leishmania

The UPR in Leishmania species plays a critical role in enabling these kinetoplastid parasites to survive and thrive within the harsh intracellular environments of host macrophages [78,79,80,81]. Upon infection, Leishmania encounters a myriad of stressors in the parasitophorous vacuole, including oxidative stress, nutrient deprivation, and immune pressures. The UPR serves as an adaptive mechanism, maintaining proteostasis and modulating host–parasite interactions, thus underpinning parasite survival and establishing its pathogenesis. Molecular chaperones, particularly members of the heat shock protein 70 (HSP70) family, are central to the Leishmania UPR. These chaperones mitigate protein aggregation, assist in protein folding, and support endoplasmic reticulum-associated degradation (ERAD), ensuring the clearance of misfolded or damaged proteins. Notably, the unique structural motifs of Leishmania HSP70 proteins render them amenable to selective inhibition, providing a promising avenue for therapeutic interventions aimed at disrupting parasite proteostasis without harming host cells. Beyond its intrinsic proteostasis machinery, Leishmania actively modulates host UPR signalling to reinforce its intracellular niche. Studies on L. amazonensis have demonstrated the activation of the IRE1-XBP1 branch of the UPR during infection. This pathway mitigates cellular oxidative stress and upregulates interferon beta (IFN-1β), a key factor facilitating the establishment of infection and pathogenesis [36]. IFN-1β induction is mediated via Toll-like receptor 2 (TLR2), a pattern recognition receptor, and has been shown to promote L. amazonensis growth [82]. The silencing of XBP1 reduces infection by suppressing IFN-1β expression, while also decreasing heme oxygenase (HO) levels and increasing nitric oxide (NO) concentrations, further highlighting XBP1’s critical role in parasite proliferation within macrophages [80]. The PERK-eIF2α-ATF4 axis of the UPR is another essential pathway leveraged by Leishmania. Infection with L. amazonensis induces significant PERK activation as early as 8 h post-infection, with levels comparable to those observed in thapsigargin-treated positive controls. PERK activation attenuates global protein synthesis while enhancing stress-response gene expression, creating a favourable intracellular environment for parasite survival. Experiments with PERK or ATF4 knockdown in infected macrophages have demonstrated a marked reduction in parasite burden, reinforcing the importance of this pathway in infection maintenance [81]. Moreover, PERK activation phosphorylates NRF2, leading to the induction of antioxidant genes that protect macrophages from oxidative stress [35,81]. Further studies have shown that Leishmania exploits host apoptotic pathways through UPR modulation. By inhibiting mitochondrial cytochrome c release and suppressing caspase activation, Leishmania delays macrophage apoptosis, thereby extending the duration of its replicative niche. Anti-apoptotic proteins such as Bcl-2 are upregulated during infection, facilitating parasite persistence. Conversely, under certain conditions, the parasite may promote controlled apoptosis to facilitate dissemination to uninfected cells, showcasing the dynamic interplay between the parasite and host cell death pathways. The UPS of Leishmania also plays a critical role in its adaptive survival strategy. Specialized components, including unique E3 ligases and deubiquitinating enzymes, characterize this system, enabling the efficient degradation of misfolded proteins. Targeting Leishmania-specific UPS components with proteasome inhibitors has shown promise in preclinical models, inducing proteotoxic stress and significantly impairing parasite viability. Distinct Leishmania species exhibit variations in UPR modulation, which may reflect differences in their pathogenic mechanisms. Studies on L. infantum demonstrated a mild yet significant induction of UPR markers, including sXBP1 and GRP78/HSPA5 proteins, in infected macrophages, while phospho-eIF2α and DDIT3/CHOP were not significantly induced [79]. This selective attenuation of host UPR pathways delays macrophage apoptosis, allowing the parasite to sustain its intracellular environment. Similarly, L. donovani infection in the Indian subcontinent has been shown to induce the UPR via PERK-dependent mechanisms. This activation delays macrophage apoptosis and promotes the upregulation of inhibitors of apoptosis proteins (cIAP1 and cIAP2), further enhancing parasite survival [83]. The activation of UPR pathways in infected macrophages not only promotes parasite survival but also impacts disease outcomes in humans. For instance, RNA samples from skin lesions of L. braziliensis-infected patients with cutaneous leishmaniasis revealed elevated ATF4 expression compared with healthy controls, highlighting the role of the PERK/eIF2α/ATF4 axis in human infection [80,81]. The interplay between the IRE1-XBP1 and PERK-eIF2α branches underscores the multifaceted nature of the UPR in Leishmania pathogenesis. In summary, the UPR in Leishmania represents a sophisticated survival strategy that integrates proteostasis maintenance, host–pathogen interaction modulation, and apoptotic regulation. Targeting Leishmania-specific UPR components, such as HSP70 proteins, the PERK-eIF2α-ATF4 axis, and UPS elements, offers a compelling approach for the development of innovative antileishmanial therapies. These strategies hold the potential to disrupt parasite survival mechanisms while preserving host cell integrity, paving the way for transformative advances in the treatment of leishmaniasis.

## 4. Broader Implications of Parasite-Induced Proteinopathies

### 4.1. Plasmodium Species

The study of parasite-induced proteinopathies, particularly those caused by Plasmodium species, offers profound insights that extend far beyond the realm of infectious diseases. Cerebral malaria (CM), a severe complication of Plasmodium falciparum infection, shares striking molecular and pathological similarities with neurodegenerative disorders such as Alzheimer’s and Parkinson’s diseases (Table 3). These parallels not only enhance our understanding of CM but also provide a unique lens through which to explore the mechanisms underlying non-parasitic proteinopathies. Furthermore, the identification of common molecular pathways and biomarkers could revolutionize diagnostic approaches for a wide range of diseases. One of the most compelling findings from the study of Plasmodium-induced proteinopathies is the overlap in molecular pathways between CM and neurodegenerative diseases. For instance, genes such as SNCA (encoding α-synuclein), SIAH2, HSPA1A (encoding HSP-70), and PINK1 are upregulated in children with CM, mirroring the molecular dysregulation observed in Parkinson’s and Alzheimer’s diseases [84,85]. α-Synuclein, a protein predominantly expressed in the brain, plays a central role in the pathogenesis of Parkinson’s disease, where its aggregation leads to neuronal dysfunction and death [86]. The overexpression of SNCA in CM suggests that α-synuclein aggregation may contribute to the neurological damage observed in severe malaria. This finding not only highlights a potential pathogenic mechanism in CM but also provides a model for studying the role of α-synuclein in other neurodegenerative conditions. By understanding how Plasmodium infection triggers α-synuclein overexpression and aggregation, researchers can gain insights into the molecular processes driving proteinopathies in non-infectious diseases. The UPS, a critical regulator of protein homeostasis, is another area of intersection between CM and neurodegenerative disorders. In CM, the dysregulation of UPS components, such as the upregulation of UBB and the downregulation of UBD and PSMC5, closely resembles the UPS dysfunction observed in Alzheimer’s and Parkinson’s diseases [87,88]. The UPS is responsible for degrading misfolded and aggregated proteins, and its impairment leads to the accumulation of toxic protein aggregates, a hallmark of many neurodegenerative disorders. By studying how Plasmodium infection disrupts the UPS, researchers can uncover mechanisms of protein aggregation and degradation that are relevant to a broad spectrum of proteinopathies. This knowledge could inform the development of diagnostic tools aimed at detecting early signs of protein homeostasis disruption, which could be beneficial for both infectious and non-infectious diseases. Molecular chaperones, such as HSP-70, also play a critical role in both CM and neurodegenerative diseases. HSP-70 is known to inhibit the aggregation of α-synuclein and other misfolded proteins, but its effectiveness depends on various factors, including nucleotide binding and the presence of co-chaperones [89]. In CM, the overexpression of HSPA1A may reflect an attempt to counteract the accumulation of misfolded proteins, similar to what is observed in neurodegenerative diseases. This parallel suggests that molecular chaperones could serve as biomarkers for protein misfolding and aggregation in both CM and neurodegenerative disorders. For example, elevated levels of HSP-70 in blood or cerebrospinal fluid could indicate ongoing protein stress, providing a measurable indicator of disease severity or progression. The identification of common molecular pathways between CM and neurodegenerative diseases also opens up new possibilities for biomarker discovery. Transcriptomic analyses of peripheral blood mononuclear cells (PBMCs) from children with CM have revealed significant changes in the expression of genes involved in protein homeostasis, inflammation, and oxidative stress [90]. These changes, particularly the upregulation of SNCA, SIAH2, HSPA1A, and PINK1, could serve as biomarkers for the severity of CM and the risk of neurological sequelae. For example, the overexpression of SNCA and SIAH2 in CM patients suggests that these genes could be used to predict the likelihood of long-term neurological damage, such as epilepsy or cognitive impairment, in survivors of severe malaria [91]. The potential of these biomarkers extends beyond CM. Given the similarities in gene expression profiles between CM and neurodegenerative diseases, the same biomarkers could be explored for early diagnosis and prognosis in conditions like Parkinson’s and Alzheimer’s diseases. For instance, the upregulation of PINK1, a mitochondrial kinase involved in cell survival under oxidative stress, has been observed in both CM and neurodegenerative disorders [92]. This suggests that PINK1 levels in blood or cerebrospinal fluid could serve as a biomarker for mitochondrial dysfunction, a common feature of many neurodegenerative diseases. Similarly, the dysregulation of the UPS, as indicated by changes in UBB, UBD, and PSMC5 expression, could provide a measurable indicator of protein homeostasis disruption in both infectious and non-infectious proteinopathies. In conclusion, the study of Plasmodium-induced proteinopathies, particularly in the context of CM, provides valuable insights into the molecular mechanisms underlying protein homeostasis dysregulation. The parallels between CM and neurodegenerative diseases, such as Parkinson’s and Alzheimer’s, highlight the potential for cross-disciplinary research that could lead to the identification of novel biomarkers. By leveraging the similarities in molecular pathways, researchers can develop new diagnostic tools that are applicable to a wide range of diseases, ultimately improving outcomes for patients with both infectious and non-infectious proteinopathies.

### 4.2. Toxoplasma gondii

The broader implications of parasite-induced proteinopathies, particularly those caused by Toxoplasma gondii, extend far beyond the immediate effects of the infection itself. Understanding the mechanisms by which Toxoplasma induces neurodegeneration and protein dysregulation can provide critical insights into the pathogenesis of other neurodegenerative diseases, such as Alzheimer’s and Parkinson’s disease. These conditions, like chronic Toxoplasma infection, are characterized by neuroinflammation, synaptic loss, and neuronal cell death, suggesting that the pathways activated by the parasite may overlap with those involved in non-parasitic proteinopathies. For instance, the activation of microglia and the complement system, which are central to the neurodegenerative processes observed in Toxoplasma-infected brains, are also key players in Alzheimer’s disease [93,97]. The upregulation of complement proteins such as C1q and C3, as well as the recruitment of activated microglia, mirrors the neuroinflammatory responses seen in Alzheimer’s pathology, where these components are implicated in synaptic pruning and neuronal damage [94,97]. This parallel suggests that studying Toxoplasma-induced neurodegeneration could reveal shared molecular pathways that drive neuronal loss in both infectious and non-infectious contexts. Moreover, the specific regions of the brain affected by Toxoplasma infection, such as the anterior cingulate cortex (ACC) and somatomotor cortex (SC), are also implicated in cognitive and motor functions that are disrupted in Alzheimer’s and Parkinson’s disease. The selective vulnerability of these regions to Toxoplasma-induced damage raises questions about whether similar mechanisms of regional susceptibility are at play in other neurodegenerative diseases. For example, the ACC is involved in conflict monitoring and error detection, functions that are often impaired in Alzheimer’s patients, while the SC is critical for motor control, which is compromised in Parkinson’s disease [96,98]. The fact that Toxoplasma infection leads to neurodegeneration in these areas suggests that the parasite may be exploiting or exacerbating pre-existing vulnerabilities in the brain, which could also be relevant to understanding how non-parasitic proteinopathies target specific neuronal populations. Another significant implication of Toxoplasma-induced proteinopathies is the potential for identifying novel biomarkers of neurodegeneration. The upregulation of specific proteins, such as CX3CL1 (fractalkine), in degenerating neurons provides a potential marker for the early detection of neuronal damage [99,100]. CX3CL1, which is expressed by neurons and binds to the CX3CR1 receptor on microglia, plays a crucial role in mediating neuron-microglia communication and is implicated in the clearance of damaged neurons [99]. In Toxoplasma-infected brains, the overexpression of CX3CL1 in degenerating neurons, coupled with the accumulation of activated microglia, suggests that this chemokine could serve as a biomarker for ongoing neuroinflammatory processes. Similarly, the deposition of complement proteins such as C1q and C3 on degenerating neurons could provide another set of biomarkers for neurodegeneration, as these proteins are known to tag dysfunctional neurons for removal by microglia [94,101]. These findings could be extrapolated to other neurodegenerative diseases where similar complement-mediated mechanisms are at play, offering new avenues for the early diagnosis and monitoring of disease progression. The role of Toxoplasma in disrupting glutamate and GABA signaling further underscores the broader implications of parasite-induced proteinopathies. Both glutamatergic and GABAergic neurons are affected by chronic Toxoplasma infection, leading to alterations in synaptic plasticity and neuronal connectivity [102,103]. These disruptions are not unique to Toxoplasma; similar changes in glutamate and GABA signaling are observed in schizophrenia, Alzheimer’s disease, and Parkinson’s disease. For example, altered glutamate signaling has been linked to cognitive deficits in Alzheimer’s disease and motor dysfunction in Parkinson’s disease, while disruptions in GABAergic signaling are associated with seizures and anxiety disorders [102,104]. The fact that Toxoplasma infection induces similar changes in neurotransmitter systems suggests that the parasite could serve as a model for studying the broader consequences of synaptic dysfunction in neurodegenerative diseases. By elucidating how Toxoplasma disrupts these pathways, researchers may gain insights into the mechanisms underlying synaptic loss and neuronal dysfunction in other proteinopathies. Furthermore, the chronic nature of Toxoplasma infection, which persists for the lifetime of the host, provides a unique opportunity to study the long-term effects of sustained neuroinflammation on the brain. The low-grade inflammation observed in Toxoplasma-infected brains, characterized by microglial activation and increased levels of cytokines and chemokines, mirrors the chronic inflammatory state seen in aging brains and in neurodegenerative diseases [95,105]. This persistent inflammation is thought to contribute to the progressive nature of diseases like Alzheimer’s and Parkinson’s, where neuroinflammatory processes exacerbate neuronal damage over time [93,105]. By studying how Toxoplasma maintains this inflammatory state and how it leads to neurodegeneration, researchers may uncover new mechanisms by which chronic inflammation drives neuronal loss in other contexts. In addition to providing insights into disease mechanisms, the study of Toxoplasma-induced proteinopathies could also inform the development of new therapeutic strategies. For example, the identification of complement proteins and CX3CL1 as key players in Toxoplasma-induced neurodegeneration suggests that targeting these pathways could be beneficial in other neurodegenerative diseases. While this paragraph focuses on the broader implications rather than therapeutic targets, it is worth noting that the insights gained from studying Toxoplasma could have far-reaching consequences for understanding and treating a wide range of neurological disorders. In conclusion, the study of Toxoplasma gondii-induced proteinopathies offers a unique window into the mechanisms of neurodegeneration and neuroinflammation that are relevant to a broad spectrum of neurological diseases. By exploring the parallels between Toxoplasma infection and non-parasitic proteinopathies, researchers can uncover shared pathways of neuronal damage, identify novel biomarkers, and gain a deeper understanding of the long-term effects of chronic neuroinflammation. These insights not only enhance our understanding of Toxoplasma infection but also contribute to the broader field of neurodegenerative disease research, potentially leading to new diagnostic tools and therapeutic approaches.

### 4.3. Leishmania Species

The broader implications of parasite-induced proteinopathies, particularly those caused by Leishmania species, extend beyond the immediate effects of the infection and offer valuable insights into the mechanisms underlying non-parasitic proteinopathies such as Alzheimer’s and Parkinson’s disease. Leishmania, an intracellular parasite, induces significant changes in host cell protein homeostasis, leading to protein misfolding, aggregation, and the dysregulation of cellular pathways. These processes share striking similarities with the pathological mechanisms observed in neurodegenerative diseases, where protein misfolding and aggregation are central to disease progression. For instance, Leishmania infection has been shown to disrupt host cell autophagy, a critical process for the clearance of misfolded proteins and damaged organelles [106]. Autophagy dysfunction is also a hallmark of neurodegenerative diseases, where the impaired clearance of aggregated proteins like tau and α-synuclein contributes to neuronal cell death [107,108]. By studying how Leishmania manipulates autophagy and other protein quality control mechanisms, researchers can gain a deeper understanding of the molecular pathways that go awry in neurodegenerative conditions. This intersection between parasitic infections and neurodegenerative diseases highlights the potential for cross-disciplinary insights that could inform the development of novel therapeutic strategies for both infectious and non-infectious proteinopathies. Moreover, Leishmania-induced proteinopathies may provide a unique model for studying the role of inflammation in protein misfolding and aggregation. The parasite triggers a robust inflammatory response in host cells, characterized by the activation of microglia and the release of pro-inflammatory cytokines [109,110]. Chronic inflammation is a common feature of neurodegenerative diseases, where it exacerbates neuronal damage and contributes to disease progression. For example, the activation of microglia and the release of inflammatory mediators have been implicated in the pathogenesis of Alzheimer’s disease and Parkinson’s disease [111,112]. By examining how Leishmania infection induces and sustains inflammation, researchers can identify key molecular players that may also be involved in the inflammatory processes driving neurodegeneration. This could lead to the discovery of novel biomarkers for the early detection and monitoring of neurodegenerative diseases. In addition to inflammation, Leishmania infection also disrupts mitochondrial function, leading to oxidative stress and the accumulation of damaged mitochondria. Mitochondrial dysfunction is a well-established feature of neurodegenerative diseases, where it contributes to neuronal cell death and the progression of pathology [113,114]. The parasite’s ability to impair mitochondrial quality control mechanisms, such as mitophagy, mirrors the mitochondrial defects observed in Parkinson’s disease, where mutations in genes like PINK1 and PRKN disrupt mitophagy and lead to the accumulation of damaged mitochondria [115,116]. By studying how Leishmania induces mitochondrial dysfunction, researchers can uncover new insights into the mechanisms underlying mitochondrial pathology in neurodegenerative diseases. This could lead to the identification of novel biomarkers for mitochondrial dysfunction, which could be used to track disease progression and response to treatment in patients with neurodegenerative conditions. Another important implication of Leishmania-induced proteinopathies is the potential for identifying novel biomarkers of neurodegeneration. The parasite’s ability to induce protein misfolding and aggregation in host cells provides a unique opportunity to study the molecular signatures associated with these processes. For example, Leishmania infection has been shown to upregulate HSPs in host cells, which play a critical role in protein folding and stress responses. HSPs are also implicated in neurodegenerative diseases, where they are involved in the clearance of misfolded proteins and the protection of neurons from stress-induced damage [117,118]. By studying the expression patterns of HSPs and other stress response proteins in Leishmania-infected cells, researchers can identify potential biomarkers for protein misfolding and aggregation that may also be relevant to neurodegenerative diseases. These biomarkers could be used to develop new diagnostic tools for the early detection of neurodegenerative conditions, as well as to monitor disease progression. Furthermore, Leishmania infection provides a unique model for studying the role of immune responses in proteinopathies. The parasite induces a robust adaptive immune response in host cells, characterized by the activation of T cells and the production of autoantibodies against parasite-derived antigens [119,120]. Interestingly, similar immune responses have been observed in neurodegenerative diseases, where T cells and autoantibodies target misfolded proteins like α-synuclein and tau [119,120,121]. By studying how Leishmania infection triggers immune responses against misfolded proteins, researchers can gain new insights into the mechanisms underlying autoimmune responses in neurodegenerative diseases. This could lead to the identification of novel biomarkers for autoimmune activity, which could be used to track disease progression and response to immunomodulatory therapies in patients with neurodegenerative conditions. In conclusion, the study of Leishmania-induced proteinopathies offers valuable insights into the mechanisms underlying non-parasitic proteinopathies such as Alzheimer’s and Parkinson’s disease. By examining how Leishmania disrupts protein homeostasis, induces inflammation, impairs mitochondrial function, and triggers immune responses, researchers can identify novel biomarkers for neurodegenerative diseases. These cross-disciplinary insights highlight the potential for leveraging knowledge from parasitic infections to advance our understanding of neurodegenerative conditions and develop new strategies for their diagnosis and treatment.

## 5. Conclusions

In conclusion, the study of parasite-induced proteinopathies illuminates a critical nexus between chronic, multi-parasitic infections and the molecular underpinnings of neurodegenerative diseases. Parasites such as Leishmania, Plasmodium, and Toxoplasma selected for their global health impact, diverse host manipulation strategies, and propensity to induce chronic or latent infections serve as powerful models to unravel how pathogens disrupt proteostasis. Chronic infections, including visceral leishmaniasis, recrudescent malaria, and lifelong Toxoplasma encephalitis, exemplify the insidious nature of parasitosis, where persistent immune activation, cumulative proteotoxic stress, and unresolved inflammation mirror pathways central to neurodegenerative disorders like Alzheimer’s and Parkinson’s diseases. These pathogens are emblematic of broader challenges in neglected tropical diseases (NTDs), where overlapping infections in endemic regions create a “syndemic” burden, amplifying organ damage, cognitive decline, and socioeconomic disparities. For instance, co-infections with Plasmodium and Leishmania in regions like East Africa or South Asia exacerbate anemia, hepatic dysfunction, and immune exhaustion, while Toxoplasma’s latent brain cysts may synergize with other neurotropic pathogens to accelerate neurodegeneration. The selection of these parasites is strategic: Leishmania disrupts lysosomal–autophagy pathways, Plasmodium induces endoplasmic reticulum (ER) stress and oxidative damage, and Toxoplasma hijacks host transcriptional machinery, collectively offering a mosaic of mechanisms to study proteostatic collapse. Their chronicity and adaptability underscore the urgent need for diagnostics and therapies that address not only acute parasitemia but also the lingering proteopathic sequelae that fuel long-term morbidity. Looking ahead, broader clinical prospects demand a paradigm shift toward holistic, systems-level approaches to combat chronic and multi-parasitic diseases. First, diagnostic innovation must evolve beyond pathogen detection to quantify proteostatic dysfunction, for example, portable mass spectrometry devices to profile protein aggregates in blood or cerebrospinal fluid, or CRISPR-based assays to identify parasite-specific exosomes that modulate host proteostasis. Such tools could differentiate mono- from co-infections and stratify patients by risk of chronic complications, such as Plasmodium-induced cognitive impairment or Leishmania-associated hepatic amyloidosis. Second, therapeutic development should prioritize dual-action agents that target both parasitic survival mechanisms and host proteostatic recovery. Examples include repurposing HSP90 inhibitors to disrupt parasite chaperones while enhancing host protein refolding, or designing autophagy-inducing nanoparticles that clear intracellular parasites and α-synuclein aggregates simultaneously. Third, longitudinal cohort studies in endemic regions are essential to map how multi-parasitic infections drive comorbidities such as the interplay between Toxoplasma-induced neuroinflammation and HIV-associated neurodegeneration or how malnutrition and parasitic burden synergistically impair proteostatic resilience in children. Moreover, translational opportunities lie at the intersection of parasitology and neurodegenerative research. Global health equity must also guide these efforts: affordable, point-of-care diagnostics and heat-stable therapeutics are critical for low-resource settings where chronic parasitosis is endemic. Finally, policy and education initiatives are needed to reframe chronic parasitosis as a determinant of non-communicable diseases (NCDs), advocating for integrated NTD-NCD programs in global health agendas. By dismantling silos between tropical disease and neurology, this research underscores a unifying truth: the fight against parasites is inseparable from the quest to resolve proteinopathies. Cross-disciplinary collaboration spanning parasitologists, neuroscientists, bioengineers, and community health experts will be pivotal in translating these insights into scalable solutions. From CRISPR-edited probiotics that restore gut proteostasis in Leishmania patients, to blockchain-enabled supply chains for distributing protease inhibitors in malaria-endemic zones, the future of this field lies in innovation that is as adaptable and resilient as the parasites themselves. By confronting the dual scourge of chronic parasitosis and neurodegeneration, we not only address pressing global health inequities but also unlock fundamental insights into the biology of aging, immunity, and cellular resilience, ultimately paving the way for a new era of precision medicine.

## Figures and Tables

**Table 1:** Summary of Parasite-Induced Proteinopathies and Their Mechanisms.

Parasite	Key Mechanisms of Protein Misfolding	Clinical Outcomes	Therapeutic Targets
*Plasmodium* spp.	- Export of virulence factors (e.g., PfEMP1) [21] - Induction of oxidative stress [22] - Disruption of calcium homeostasis [9,11]	Cerebral malaria, placental malaria	- HSP70 inhibitors - Proteasome enhancers - Autophagy modulators
*Toxoplasma gondii*	- Secretion of dense granule proteins (GRAs) [9] - Manipulation of host UPR and autophagy [23,24] - Disruption of calcium homeostasis [9,11]	Neurodegeneration, chronic toxoplasmosis	- TgHSP70 inhibitors - PERK-like kinase inhibitors - Autophagy modulation
*Leishmania* spp.	- Secretion of exosomes containing HSP70/90 [3] - Induction of oxidative stress [13] - Disruption of autophagy [15]	Cutaneous, mucocutaneous, visceral leishmaniasis	- HSP70 inhibitors - PERK/elF2α pathway inhibitors - Proteasome inhibitors

**Table 2:** Therapeutic Agents Targeting Parasite-Induced Proteinopathies.

Therapeutic Agent	Target	Mechanism of Action	Potential Applications
Violacein	HSP70/90 in Plasmodium	Disrupts parasite proteostasis, leading to protein aggregation and parasite death	Malaria [21,32,33]
PK4 Inhibitors	PK4 kinase in Plasmodium	Inhibits parasite-specific UPR, reducing parasite survival under stress	Malaria [34]
TgHSP70 Inhibitors	TgHSP70 in Toxoplasma	Disrupts protein folding and ERAD, impairing parasite replication	Toxoplasmosis [10,35]
Proteasome Inhibitors	Leishmania-specific UPS components	Induces proteotoxic stress, leading to parasite death	Leishmaniasis [21,36]
Autophagy Enhancers	Host autophagy pathways	Enhances clearance of misfolded proteins, reducing parasite burden	Multiple parasitic infections and neurodegenerative diseases [16,26,31]

**Table 3:** Potential Biomarkers for Parasite-Induced Proteinopathies and Neurodegenerative Diseases.

Biomarker	Role in Parasite Infections	Role in Neurodegenerative Diseases
α-Synuclein (SNCA)	Upregulated in cerebral malaria, contributing to neuronal damage [80]	Aggregates in Parkinson’s disease, leading to neuronal dysfunction [81]
HSP70 (HSPA1A)	Overexpressed in response to protein misfolding in malaria and toxoplasmosis [10,58]	Protects against protein aggregation in Alzheimer’s and Parkinson’s [89]
PINK1	Upregulated in cerebral malaria, indicating mitochondrial stress [93]	Mutations linked to mitochondrial dysfunction in Parkinson’s disease [94]
CX3CL1 (Fractalkine)	Overexpressed in Toxoplasma-induced neurodegeneration, mediating neuron-microglia communication [95]	Implicated in neuroinflammation in Alzheimer’s disease [96]
